# Death of Mr. Vance

**Published:** 1837-07-01

**Authors:** 


### 6. Death of Mr. Vance.

The Medical World is too well acquainted with the violent and untimely end of Mr. Vance, to render it necessary for us to go at length into the particulars. We would willingly, indeed, be spared the painful office of chronicling the death of an old and esteemed friend.

Mr. Vance entered the medical department of the Navy in 1792, and was promoted to be a full surgeon two years after. He then served at sea for twelve or fourteen years, when he was appointed one of the surgeons of Haslar, the great naval hospital. Here he acquired the reputation of a good operative surgeon ; but, in consequence of some differences which occurred, he, as well as several other surgeons, were removed ; and his next service was in a small hospital at Painton, in Devonshire, where he married. At the peace of 1815, Mr. Vance was restored to his old quarters at Haslar, from whence he was once more removed to the charge of Greenwich Hospital in 1819. Soon after this he was allowed to retire on the invalid list, and settled in London.

Mr. Vance obtained a large and lucrative practice, which was rather of a medical than a surgical complexion. His reputation among his old brother officers, as well as in the fashionable circles of London stood high, higher, perhaps, than among his professional brethren. He was more remarkable for his tact than talent, more of a practical than of a scientific or a philosophic mind. He never contributed by any published papers to the advance of medicine, and his chief merit in his early years was operative dexterity, in his latter years his management of dyspeptic disorders.

On the 21st of March, being then in his 67th year he was thrown down by a madman, and received a severe scalp-wound. Erysipelas of the scalp ensued, and he died in the course of a week.

